# Prognostic significance of the expression of GFRα1, GFRα3 and Syndecan-3, proteins binding ARTEMIN, in mammary carcinoma

**DOI:** 10.1186/1471-2407-13-34

**Published:** 2013-01-26

**Authors:** Zheng-Sheng Wu, Vijay Pandey, Wen-Yong Wu, Shan Ye, Tao Zhu, Peter E Lobie

**Affiliations:** 1 Hefei National Laboratory for Physical Sciences at Microscale and School of Life Sciences, University of Science and Technology of China, Hefei, Anhui, People's Republic of China; 2 Department of Pathology, Anhui Medical University, Hefei, Anhui, People's Republic of China; 3 Cancer Science Institute of Singapore and Department of Pharmacology, National University of Singapore, Centre for Life Sciences, #03-06C, 28 Medical Drive, Singapore, 117456, Singapore; 4 Department of General Surgery, First Affiliated Hospital of Anhui Medical University, Anhui, Hefei, Anhui, People's Republic of China; 5 National Cancer Science Institute of Singapore, National University Health system, Singapore, Singapore

**Keywords:** ARTN, GFRα1, GFRα3, SDC3, Mammary carcinoma, Survival

## Abstract

**Background:**

Artemin (ARTN) has been implicated in promoting oncogenicity, tumor growth and invasiveness in diverse human malignancies. However, the clinical and prognostic significance of upstream ligand binding components, potentially mediating ARTN oncogenicity, largely remain to be determined.

**Methods:**

We determined the mRNA and protein expression of three proteins demonstrated to bind ARTN, namely GFRα1, GFRα3 and Syndecan-3 (SDC3), in benign breast disease and mammary carcinoma by *in situ* hybridization and immunohistochemistry, respectively. Their prognostic significance combined with ARTN expression was also investigated in mammary carcinoma.

**Results:**

The expression of GFRα1 and GFRα3, but not SDC3, was significantly increased in mammary carcinoma and positively associated with tumor lymph node metastases, higher clinical stage and HER-2 positivity. Moreover, both GFRα1 and GFRα3 expression were significantly associated with survival outcome of patients with mammary carcinoma by univariate and multivariate analyses, whereas expression of SDC3 was not. Co-expression of ARTN with either GFRα1 or GFRα3, but not SDC3, produced synergistic increases in the odds ratio for both relapse-free and overall survival in patients with mammary carcinoma. Furthermore, significant association of GFRα1 and GFRα3 expression with survival outcome observed herein were restricted to ER negative or HER-2 negative mammary carcinoma.

**Conclusions:**

The expression of GFRα1 and/or GFRα3, especially when combined with ARTN expression, may be useful predictors of disease progression and outcome in specific subtypes of mammary carcinoma.

## Background

Artemin (ARTN) is a growth factor belonging to the glial cell line-derived neurotrophic factor (GDNF) family of ligands (GFL) comprised of 4 members including GDNF, neurturin and persephin. In addition to its described neurotrophic role [1-3], ARTN has also been implicated in promoting oncogenicity, tumor growth and invasiveness in diverse human malignancies, including mammary, endometrial, esophageal, lung and pancreatic carcinoma [4-10].

In mammary carcinoma (MC), increased expression of ARTN has been observed compared to normal tissue and expression of ARTN in MC predicted residual disease after chemotherapy, metastasis, relapse, and death [5]. It has been reported that forced expression of ARTN promotes tumor growth by increased proliferation and survival [5,7,8]. Furthermore, ARTN promotes epithelial to mesenchymal transition and angiogenesis and enhances cancer stem cell like behaviour in ER-negative MC (ER-MC) carcinoma cells resulting in metastatic dissemination [5,11-13]. Moreover increased ARTN expression predicts poor survival of patients with ER-positve MC (ER + MC) treated with tamoxifen and forced expression of ARTN produces anti-estrogen resistance [14]. The downstream signaling pathways by which ARTN promotes cell survival, oncogenicity, drug resistance [6,7,14] and metastases [11] have been reported. However, the prognostic significance of upstream ligand binding components, potentially mediating ARTN oncogenicity in mammary carcinoma, remain to be determined.

GFL family members were initially thought to signal via high affinity preferential interaction with one or more of the GDNF receptor α family (GFRα) comprising GFRα1-4 [1-3]. The GFL- GFRα complex then binds to and activates the transmembrane RET receptor tyrosine kinase [4] which propagates cellular signaling. However, GFLs are promiscuous and interact with multiple GFRα family members, ARTN having been reported to bind and activate both GFRα1 and GFRα3 [3]. Moreover, GFLs have been reported to bind to and/or activate distinct non-GFRα proteins [15] and to function by both RET dependent and independent mechanisms [4,16,17]. Recently ARTN, as well as GDNF, has been reported to activate signaling through c-Src by binding to Syndecan-3 (SDC3) [18]. Increased GFRα1 expression has been previously reported in MC and its expression is associated with certain clinicopathologic features such as lymph node metastases [4]. However, no correlation of expression with survival outcome of patients was determined. To date, the expression and prognostic significance of GFRα3 and SDC3, the two other receptor proteins binding ARTN in MC has not been reported.

In an attempt to determine which of the ARTN binding proteins identified to date may mediate the effects of ARTN in MC, we examined the mRNA and protein expression of GFRα1, GFRα3 and SDC3 in MC and examined the correlation of expression to clinicopathologic features and patient survival outcome, both by univariate and multivariate analyses. Moreover, we correlated the combined expression of ARTN and the various receptors with patient survival outcome to determine which combination of ligand and receptor may represent the functional complex mediating mammary neoplastic progression.

## Methods

### Patients and specimens

The patient population consisted of 159 consecutive MC patients and 26 consecutive patients with benign breast disease (BBD) who underwent surgery at the First Affiliated Hospital of Anhui Medical University (Hefei, Anhui, People’s Republic of China) between 2001 and 2002. The details of this cohort have previously been described in detail [5,19] including the definition of human epidermal growth factor receptor-2 (HER-2) status according to the ASCO/CAP HER-2 Guideline Recommendations [20]. Patients with BBD include 10 cases of fibroadenoma and 16 cases of adenosis. In MC patients, there are 150 cases of invasive ductal carcinoma, 6 cases of invasive lobular carcinoma and 3 cases of mucinous carcinoma. Among 159 MC patients, 126 patients were followed for a median follow-up time of 60 months (range 8–64 months). The protocol for the use of patient samples in this study was approved by the Institutional Review Board and patient consent forms were obtained from all patients in accordance with the Declaration of Helsinki.

### Tissue microarrays (TMA) Construction

Paraffin-embedded BBD and MC specimens were obtained from archive of the Department of Pathology, the First Affiliated Hospital of Anhui Medical University, P.R. China. TMAs were constructed as previously described [21]. Three tissue “spots” from two different paraffin blocks of each case of BBD and MC were included per patient. The spot diameter for mammary tissue was 1 mm. A total of five TMA blocks were prepared and sectioned for *in situ* hybridization and immunohistochemical analysis.

### *In situ* hybridization (ISH)

Digoxin-labeled antisense oligonucleotide probes for GFRα1, GFRα3 and SDC3 were obtained from Boshide Biotech Co. (Wuhan, China). The probe sequences were as follows:

GFRα1

5^′^-TTCAT ATCAG ATGTT TTTCA GCAAG TGGAG CACAT-3^′^;

GFRα3

5^′^-TGCCA CCGGC GCATG AAGAA CCAGG TTGCC TGCTT-3^′^,

5^′^-CACTG CCAGC GCCAC GTCTG CCTCA GGCAG CTGCT-3^′^ and

5^′^-GATTT CCAGA CCCAC TGCCA TCCCA TGGAC ATCCT −3^′^.

SDC3

5^′^-CAGCG CTGGC GCAGT GAGAA CTTCG AGAGA CCCGT-3^′^ and

5^′^-TACTT CGAGC AGGAG TCGGG CATTG AGACA GCCAT −3^′^

ISH was performed as described previously [19,22]. Briefly, 4 μm-thick TMA sections were deparaffinized, rehydrated, and then digested with pepsin for 20 min at 37°C and refixed in 4% paraformaldehyde. After the sections were washed with PBS, hybridization solution was placed on each section for 2 h and then replaced with hybridization solution with probes (or scrambled probes for negative control samples) at 40°C for 20 h. After washing with sodium chloride-sodium citrate (SSC), the sections were incubated with an anti-digoxin antibody followed by binding to streptavidin-biotin-peroxidase complex solution. After that, the sections were stained with 3, 3´-diaminobenzidine solution and counterstained with hematoxylin solution.

### Immunohistochemistry (IHC)

Immunohistochemical analysis of GFRα1, GFRα3 and SDC3 protein expression was performed on TMA sections (4 μm thick) with polyclonal antibodies against GFRα1(1:100 dilution; Santa Cruz Biotechnologies, Santa Cruz, CA), GFRα3 (1:100 dilution; Santa Cruz Biotechnologies) and SDC3 (1:80 dilution; ProteinTech Group, Chicago, IL) by the peroxidase-conjugated streptavidin complex method (Histostain-SP Kit, Zymed, San Francisco, CA) as previously described [5,19,22].

### Review and scoring

The stained sections were reviewed and scored for expression of GFRα1, GFRα3 and SDC3 with a light microscope (Olympus American Inc., Melville, NY) independently by two pathologists without knowledge of the patient’s clinical or histopathological information as previously described [5,19,22]. The rare cases with discordant scores were re-evaluated and scored on the basis of consensual opinion. The sections were scored on the basis of the staining intensity and the percentage of cells with staining relative to the background [23]. The evaluation of extent of staining was based on the percentage of positive-stained tumor cells among all the tumor cells in each case and classified into 4 categories: 0 (0%), 1 (1%-25%), 2 (26%-50%), 3 (51%-75%), and 4 (76%-100%). The intensity of staining was based on the color intensity of the tumor cells in each case and classified into 4 categories: 0 (negative), 1 (weak), 2 (medium), and 3 (strong). The sum of the intensity and extent score was used as the final score (0–7). Tissue specimens having a final score >2 were considered positive.

### Statistical analysis

All statistical analyses were performed using SPSS software system for Windows (version 13.0; SPSS, Chicago, IL). The chi-squared (χ2) test was used to analyze the difference in the expression levels among different samples. The statistical significance of potential correlations was determined using the χ^2^ test. Pearson’s correlation coefficient was calculated to evaluate the relationships between the expression of GFRα1, GFRα3 or SDC3 and ARTN expression. Kaplan-Meier curves were constructed to determine patient relapse-free survival (RFS) and overall survival (OS) rates. Cox regression analysis was performed to determine the association of GFRα1, GFRα3 and SDC3 expression to the risk of relapse and death. The statistical differences in survival among subgroups were compared using the log-rank test. *P* values < 0.05 were considered statistically significant.

## Results

### Expression of GFRα1, GFRα3 and SDC3 mRNA and protein in benign breast disease and mammary carcinoma

**Figure 1:**
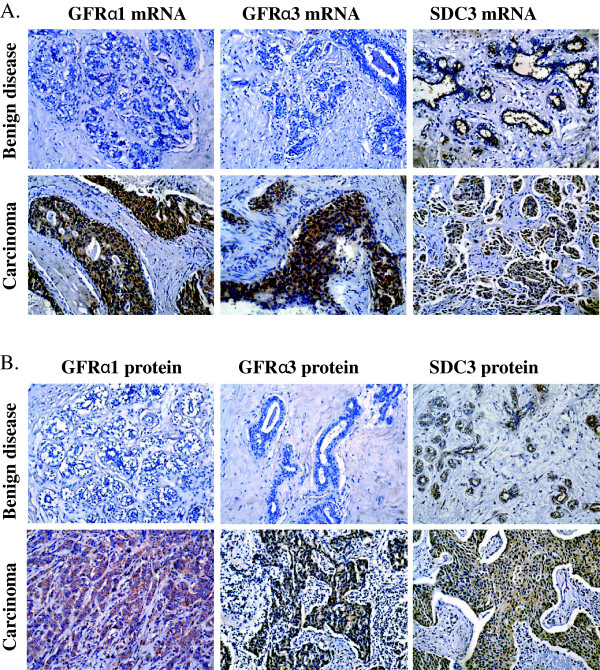
***In situ*hybridization and immunohistochemical analysis of GFRα1, GFRα3 and SDC3 mRNA and protein expression in benign breast disease and mammary carcinoma.*A****, In situ* hybridization analysis. *Up,* low expression of GFRα1 and GFRα3 mRNA and high expression of SDC3 mRNA in mammary tissue derived from patients with benign breast disease; *Bottom*, high expression of GFRα1, GFRα3 and SDC3 mRNA in mammary carcinoma. ***B****,* Immunohistochemistry. *Up,* low expression of GFRα1 and GFRα3 protein and high expression of SDC3 protein in mammary tissue derived from patients with benign breast disease; *Bottom*, high expression of GFRα1, GFRα3 and SDC3 protein in mammary carcinoma. All images are counterstained with hematoxylin. Photomicrographs were captured at 200X magnification.

We first utilized ISH to determine the expression of GFRα1, GFRα3 and SDC3 mRNA in mammary tissue from benign breast disease (BBD) and MC. GFRα1, GFRα2 and SDC3 mRNA expression was observed in 6 (23.1%), 5 (19.2%) and 9 (34.6%) of the 26 BBD tissue samples respectively. Weak or moderate expression of GFRα1 and GFRα3 mRNA was observed in the cytoplasm of epithelial cells of mammary ducts and acini. Moderate expression of SDC3 mRNA was observed in mammary tissue and similarly localized in the cytoplasm of the epithelium. In contrast to BBD, 80 (50.3%) and 68 (42.8%) of 159 MC specimens were positive for GFRα1 and GFRα3 mRNA respectively, which was a significantly higher percentage than that observed in BBD tissues (*P* = 0.010 and *P* = 0.023, Table 1). Moderate or strong expression of GFRα1 and GFRα3 mRNA was predominantly localized in the carcinoma cells with an infrequently positive signal located in stromal cells (Figure 1). As shown in Figure 1, the positive signal for SDC3 mRNA was mainly localized in cytoplasm with infrequent expression in the nuclei of carcinoma cells in MC tissue. However, the percentage expression of SDC3 mRNA was similar and non-significant between BBD and MC tissues (positive rates of 35.8% and 34.6% (*P* = 0.903) respectively, Table 1).

**Table 1:** Comparative expression of GFRα1, GFRα3 and SDC3 in benign breast disease (BBD) and mammary carcinoma (MC)

		**GFRα1 expression (n (%))**				**GFRα3 expression (n (%))**				**SDC3 expression (n (%))**			
**Group**	***n***	**mRNA**	***P***	**protein**	***P***	**mRNA**	***P***	**protien**	***P***	**mRNA**	***P***	**protein**	***P***
BBD	26	6(23.1)	**0.01**	5(19.2)	0.067	5(19.2)	**0.023**	3(11.5)	**0.037**	9 (34.6)	0.903	8(30.8)	0.796
MC	159	80(50.3)		60(37.7)		68(42.8)		50(31.4)		57(35.8)		45(28.3)	

Values in *bold* are significant (*P* < 0.05).

We next utilized IHC to determine the expression of immunoreactive protein for GFRα1, GFRα3 and SDC3 in the same cohort of specimens. Although the IHC detection appeared less sensitive than ISH, similar expression patterns of GFRα1, GFRα3 and SDC3 protein were observed in the BBD and MC tissues as for mRNA expression. Pearson’s correlation analysis demonstrated a significant association of expression of GFRα1 mRNA with both GFRα1 and GFRα3 protein and a significant association of GFRα3 mRNA with both GFRα1 and GFRα3 protein (Additional file 1: Table S1). SDC3 mRNA was significantly associated with SDC3 protein expression.

Similar to mRNA expression, GFRα1, GFRα3 and SDC3 proteins were localized in the cytoplasm of epithelial cells of mammary ducts and acini in BBD or carcinoma cells in MC. As shown in Table 1, 37.7% (n = 60) and 31.4% (n = 50) of 159 MC specimens were positive for GFRα1 or GFRα3 protein respectively, whereas only 19.2% (n=5) and 11.5% (n=3) of 26 BBD specimens were positive for GFRα1 or GFRα3 protein (*P* = 0.067 and *P* = 0.037) respectively. Meanwhile, no significant difference of SDC3 protein expression was observed between BBD and MC specimens (*P* = 0.796). The localization of ARTN protein has previously been reported in this cohort [5] and GFRα1, GFRα3 or SDC3 protein were co-expressed with ARTN in 27.7% (n = 44), 25.2% (n = 40) and 21.4% (n = 34) of MC samples respectively (Additional file 1: Table S2). In 45.9% (n = 73) of MC samples, co-expression of ARTN protein and any one of its binding proteins GFRα1, GFRα3 or SDC3 was observed (Additional file 1: Table S2). 36% (n = 57) of MC samples were either GFRα1 or GFRα3 and ARTN positive (Additional file 1: Table S2).

### Correlation between expression of GFRα1, GFRα3 and SDC3 and clinicopathologic features of mammary carcinoma

Next, we investigated for any potential association of tumor expression of mRNA or protein for GFRα1, GFRα3 and SDC3 with the clinicopathologic features of MC. As observed in Table 2, expression of GFRα1 mRNA was significantly associated with younger patient age (*P* = 0.005), tumor lymph node metastasis (LNM) (*P* = 0.013), higher clinical stage (*P* = 0.001) and HER-2 positive expression (*P* = 0.002). The expression of GFRα3 mRNA was significantly associated with younger patient age (*P* = 0.043). Significant associations were also observed between the protein expression of GFRα1 and GFRα3 and certain clinicopathologic characteristics of MC. As observed in Table 3, both the expression of GFRα1 and GFRα3 protein were significantly associated with tumor LNM (*P* = 0.001 and *P* = 0.006), higher clinical stage (*P* = 0.001 and *P* = 0.008) and HER-2 positive expression (*P* = 0.030 and *P* = 0.005) respectively. However, no significant association was observed between SDC3 mRNA or protein expression and any clinicopathologic characteristic (all *P* > 0.05).

**Table 2:** Association of tumor GFRα1, GFRα3 and SDC3 mRNA expression with clinicopathologic parameters of patients with mammary carcinoma

		**GFRα1 expression (n (%))**		**GFRα3 expression (n (%))**		**SDC3 expression (n (%))**	
**Parameter**	***n***	**mRNA**	***P***	**mRNA**	***P***	**mRNA**	***P***
**Age (years)**							
≤ 35	16	14 (87.5)	**0.005**	11 (68.8)	**0.043**	8 (50.0)	0.399
35-55	92	45 (48.9)		40 (43.5)		33 (35.9)	
> 55	51	21 (41.2)		17 (33.3)		16 (31.4)	
**Tumor size (cm)**							
≤ 2	13	6(46.2)	0.62	4 (30.8)	0.406	7 (53.8)	0.364
2 ~ 5	115	56 (48.7)		48 (417)		39 (33.9)	
> 5	31	18 (58.1)		16 (51.6)		11 (35.5)	
**Histologic type**							
Ductal	150	78(52.0)	0.142	67(44.7)	0.127	54(36.0)	0.333
Lobular	6	2(33.3)		1(16.7)		1(16.7)	
Mucinous	3	0(0)		0(0)		2(66.7)	
**Lymph node metastasis**							
0	55	20 (36.4)	**0.013**	19 (34.5)	0.235	23 (41.8)	0.481
1 ~ 3	55	28 (50.9)		24(43.6)		17 (30.9)	
>3	49	32 (65.3)		25 (51.0)		17 (34.7)	
**Grade**							
I	13	8 (61.5)	0.436	6 (46.2)	0.678	7 (53.8)	0.124
II	102	53 (52.0)		41 (40.2)		31 (30.4)	
III	44	19 (43.2)		21 (47.7)		19 (43.2)	
**Stage**							
I-II	85	32 (37.6)	**0.001**	32 (37.6)	0.162	31 (36.5)	0.861
III-IV	74	48 (64.9)		36(48.6)		26 (35.1)	
**ER status ^**							
-	94	49 (52.1)	0.582	37 (39.4)	0.297	31 (33.0)	0.364
+	65	31 (47.7)		31 (47.7)		26 (40.0)	
**PR status ^^**							
-	90	44 (48.9)	0.681	36 (40.0)	0.421	27 (30.0)	0.079
+	69	36 (52.2)		32 (46.4)		30 (43.5)	
HER-2 *							
-	121	53 (43.8)	**0.003**	47 (38.8)	0.074	44 (36.4)	0.809
+	38	27 (71.1)		21 (55.3)		13 (34.2)	

^ ER positive required at least 10% staining nuclei.

^^ PR positive required at least 10% staining nuclei.

HER-2 positive were 3+ or 2+ and FISH confirmed.

Values in *bold* are significant (*P* < 0.05).

**Table 3:** Association of tumor GFRα1, GFRα3 and SDC3 protein expression with clinicopathologic parameters of patients with mammary carcinoma

		**GFRα1 expression (n (%))**		**GFRα3 expression (n (%))**		**SDC3 expression (n (%))**	
**Parameter**	***n***	**protein**	***P***	**protein**	***P***	**protein**	***P***
**Age (years)**							
≤ 35	16	9 (56.3)	0.249	9 (56.3)	0.078	5 (31.3)	0.768
35-55	92	34 (37.0)		26 (28.3)		24 (26.1)	
> 55	51	17 (33.3)		15 (29.4)		16 (31.4)	
**Tumor size (cm)**							
≤ 2	13	6 (46.2)	0.135	4 (30.8)	0.37	5 (38.5)	0.555
2 ~ 5	115	38 (33.0)		33 (28.7)		30 (26.1)	
> 5	31	16 (51.6)		13 (41.9)		10 (32.3)	
**Histologic type**							
Ductal	150	57 (38.0)	0.142	49 (32.7)	0.352	41 (27.3)	0.313
Lobular	6	2 (33.3)		1 (16.7)		2 (33.3)	
Mucinous	3	1 (33.3)		0 (0)		2 (66.7)	
**Lymph node metastasis**							
0	55	9 (16.4)	**0.001**	9 (16.4)	**0.006**	15 (27.3)	0.977
1 ~ 3	55	24 (43.6)		19 (34.5)		16 (29.1)	
>3	49	27 (55.1)		22 (44.9)		14 (28.6)	
**Grade**							
I	13	6 (46.2)	0.657	4 (30.8)	0.906	4 (30.8)	0.788
II	102	36 (35.2)		31 (30.4)		27 (26.5)	
III	44	18 (40.9)		15 (34.1)		14 (31.8)	
**Stage**							
I-II	85	19 (22.4)	**0.001**	19 (22.4)	**0.008**	23 (27.1)	0.709
III-IV	74	41 (55.4)		31 (41.9)		22 (29.7)	
**ER status^**							
-	94	39 (41.5)	0.24	27 (28.7)	0.374	24 (25.5)	0.351
+	65	21 (32.3)		23 (35.4)		21 (32.3)	
**PR status^^**							
-	90	32 (35.6)	0.517	28 (31.1)	0.917	22 (24.4)	0.218
+	69	28 (40.6)		22 (31.9)		23 (33.3)	
HER-2 *							
-	121	40 (33.1)	**0.03**	31 (25.6)	**0.005**	33 (27.3)	0.607
+	38	20 (52.6)		19 (50.0)		12 (31.6)	

^ ER positive required at least 10% staining nuclei.

^^ PR positive required at least 10% staining nuclei.

HER-2 positive were 3+ or 2+ and FISH confirmed.

Values in *bold* are significant (*P* < 0.05).

### Correlation between GFRα1, GFRα3, SDC3 and ARTN expression

ARTN expression has also been implicated in disease progression in the same cohort of MC specimens used herein [5]. We therefore utilized correlation analysis to determine the relationship between ARTN protein expression and the expression of GFRα1, GFRα3 or SDC3 proteins in the same cohort of MC patients. As observed in Additional file 1: Table S1, Pearson’s correlation analyses revealed that the expression of ARTN protein was significantly correlated to the protein expression of GFRα3 (r_s_ = 0.208, *P* = 0.009, respectively).

### Correlation between GFRα1, GFRα3 and SDC3 expression and patient survival

**Figure 2:**
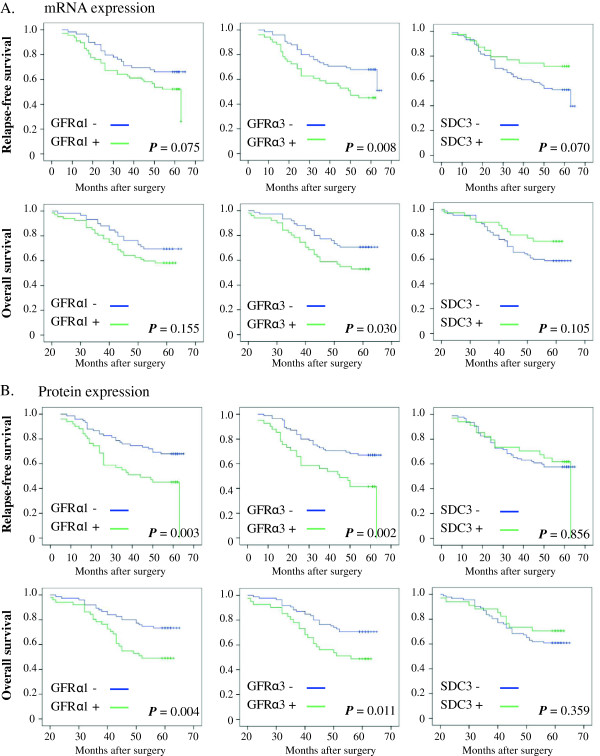
Kaplan-Meier analysis of the significance of expression of GFRα1, GFRα3 and SDC3 mRNA and protein on relapse free survival (RFS) and overall survival (OS) of patients with mammary carcinoma.

To determine the prognostic significance of GFRα1, GFRα3 and SDC3 expression in patients with MC, we firstly performed Kaplan-Meier analyses to correlate the expression of these receptors for ARTN and patient relapse free survival (RFS) and overall survival (OS). As observed in Figure 2 and Additional file 1: Table S3, patients whose tumors were positive for expression of GFRα3 mRNA exhibited a lower 5 year RFS or OS rate than patients whose tumors were negative for GFRα3 mRNA respectively (*P* = 0.008 and *P* = 0.030). Similarly, expression of GFRα3 protein also predicted a lower 5 year RFS or OS than patients whose tumors were negative for GFRα3 protein respectively (*P* = 0.002 and *P* = 0.011). Patients whose tumors expressed GFRα1 protein (but not GFRα1 mRNA) exhibited a significantly lower RFS and OS compared to patients whose tumors were negative for GFRα1 protein respectively (*P* = 0.003 and *P* = 0.004). No significant association was observed between tumor expression of SDC3 mRNA or protein and patient RFS or OS (all *P* > 0.05).

We then examined for the effect of combined expression of these receptors on RFS and OS of patients with MC. RFS and OS of patients whose tumors were negative for both GFRα1 and GFRα3 mRNA or protein were significantly higher than patients whose tumors were positive for mRNA or protein expression of both GFRα1 and GFRα3 (Additional file 1: Table S3). Moreover, the RFS and OS rates for patients whose tumors were negative for the mRNA for all the three ARTN receptors were greatly and significantly higher compared to those patients whose tumors were both GFRα1 mRNA and SDC3 mRNA negative but GFRα3 positive (*P* = 0.002 and 0.001 respectively) (Additional file 1: Table S3).

Consistent with the results of the univariate Kaplan-Meier survival analysis, multivariate analysis also revealed that the adjusted odds ratios for death or relapse of patients with MC were concordantly significantly elevated in those patients whose tumors expressed GFRα1 protein, GFRα3 mRNA or GFRα3 protein (Additional file 1: Table S4).

### Correlation between GFRα1, GFRα3 and SDC3 expression and patient survival in ER and HER2 subgroups

Given the previous reports of an association of the expression of GFRα1 and GFRα3 with ER expression [4] and tamoxifen resistance in MC [24], we further examined for a potential association of GFRα1, GFRα3 and SDC3 expression with RFS or OS in the subgroups of patients with tumors with either ER negative or ER positive expression, or with differential expression of HER-2. As shown in Additional file 1: Table S5, the expression of GFRα1 and GFRα3 protein in patients with ER positive tumors tended to correlate with RFS, but did not reach significance (*P* = 0.095 and 0.091). However, a significant positive correlation was observed between the expression of SDC3 protein and OS in patients with ER positive tumors (*P* = 0.023, Additional file 1: Table S5). In patients with ER negative tumors, the expression of either GFRα1 or GFRα3 mRNA or protein was significantly correlated with patient RFS and OS (Additional file 1: Table S6). No significant correlation was observed between SDC3 mRNA or protein expression and patient survival (all *P* > 0.05) in ER negative MC.

We next performed Kaplan-Meier analysis of the expression of the different receptors for ARTN and patient survival in the subgroups of patients with differential HER-2 expression. The expression of GFRα1 and GFRα3 protein (but not SDC3 protein) was significantly associated with decreased RFS and OS in HER-2 negative MC. The expression of GFRα3 mRNA was significantly associated with decreased RFS in HER-2 negative MC whereas the expression of SDC3 mRNA was positively and significantly associated with RFS in this subgroup (Additional file 1: Table S7). Interestingly, no significant correlation was observed between any of these three receptors for ARTN and RFS or OS in patients with HER2-positive tumors (Additional file 1: Table S8).

### Co-expression of GFRα1 or GFRα3 with ARTN predicts worse survival outcome

We next determined if co-expression of the ligand with one of the receptor proteins studied herein, rather than examination of only receptor expression, would predict a worse survival outcome for patients. Patients with tumors that expressed both ARTN and GFRα1 or ARTN and GFRα3, both by univariate and multivariate survival analysis, exhibited a worse survival outcome than patients whose tumors did not express ARTN and GFRα1 or GFRα3, suggesting that patients with tumors that were ARTN-positive and either GFRα1-positive or GFRα3-positive had a poorer outcome than any other phenotypes (Additional file 1: Table S9 and S10). Survival outcome in patients whose tumor expressed both ARTN and SDC3 was not significantly different to those patients who were negative for both proteins.

### Co-expression of receptors with ARTN is associated with a worse survival outcome in selected subgroups of mammary carcinoma

We next determined if the worse survival outcome in patients with tumors with co-expression of either GFRα1 or GFRα3 and ARTN was restricted to tumor subtypes. We therefore examined for a potential association of the expression of ARTN protein combined with GFRα1, GFRα3 or SDC3 protein expression, with RFS or OS in the subgroups of patients with tumors that are designated either ER negative or ER positive or HER-2 negative or HER-2 positive. Highly significant associations of combined ARTN and GFRα1 or GFRα3 expression with RFS or OS was observed in only the ER negative and HER-2 negative subgroups (Table 4). There was no significant association of combined ARTN and SDC3 expression with RFS or OS in the ER negative or HER-2 negative subgroups. No association of expression in any combination of protein with either RFS or OS was observed in the ER positive or HER-2 positive subgroups.

**Table 4:** Association of tumor ARTN, GFRα1, GFRα3 and SDC3 protein expression with five year relapse-free survival (RFS) and overall survival (OS) in patients with ER-positive/ER-negative or HER2-positive/HER2-negative mammary carcinoma

	**RFS (%)**	***P***	**OS (%)**	***P***
**ER-positive**				
ARTN-GFRα1-/ARTN + GFRα1+	81.0*/*50.0	0.153	90.5*/*50.0	**0.044**
ARTN-GFRα3-/ARTN + GFRα3+	81.8*/*50.0	0.199	86.4*/*50.0	0.138
ARTN- SDC3-*/*ARTN + SDC3+	54.2*/*66.7	0.679	66.7*/*66.7	0.903
**ER-negative**				
ARTN-GFRα1-/ARTN + GFRα1+	76.0*/*16.7	**0.002**	84.0*/*16.7	**0.001**
ARTN-GFRα3-/ARTN + GFRα3+	75.0*/*25.0	**0.009**	81.3*/*25.0	**0.005**
ARTN- SDC3-*/*ARTN + SDC3+	71.4*/*50.0	0.532	71.4*/*50.0	0.532
**HER2-positive**				
ARTN-GFRα1-/ARTN + GFRα1+	75.0*/*100.0	0.605	87.5*/*100.0	0.724
ARTN-GFRα3-/ARTN + GFRα3+	NA		NA	
ARTN- SDC3-*/*ARTN + SDC3+	NA		NA	
**HER2-negative**				
ARTN-GFRα1-/ARTN + GFRα1+	78.9*/*22.2	**0.001**	86.8*/*22.2	**0.001**
ARTN-GFRα3-/ARTN + GFRα3+	79.1*/*37.5	**0.01**	83.7*/*37.5	**0.005**
ARTN- SDC3-*/*ARTN + SDC3+	64.3*/*60.0	0.855	71.4*/*60.0	0.679

Note: *NA*, not available.

Values in *bold* are significant (*P* < 0.05).

## Discussion

Herein, we observed that two proteins, GFRα1 and GFRα3, previously demonstrated to bind ARTN [3], are expressed at significantly higher levels in MC compared to BBD. In contrast, the expression of a third protein, SDC3, also demonstrated to bind ARTN [18], was not increased in expression in MC. Concordantly, the expression of GFRα1 and GFRα3 was also associated with clinicopathologic features predicting a poor outcome, such as lymph node metastases and tumor stage, whereas the expression of SDC3 was not associated with any such features. Moreover, both GFRα1 and GFRα3 were associated with poor survival outcome by univariate and multivariate analyses whereas SDC3 was not. Finally, co-expression of ARTN with either GFRα1 or GFRα3 but not SDC3 produced synergistic increases in the odds ratio for both RFS and OS in patients with MC. Hence, it is apparent that GFRα1 or GFRα3 or combinations of both mediate the described oncogenic effects of ARTN in both ER negative [11] or ER positive MC [14]. Whether these observations also apply to other described ARTN sensitive cancers, such as pancreatic, endometrial and lung carcinoma [7-9,25] remains to be determined. It is also possible that further proteins that bind ARTN are yet to be identified and may also participate in the oncogenic functions of ARTN in various cancer types. Indeed, GDNF has been demonstrated to bind to and/or activate other oncogenic signaling mediators such as MET [26], N-CAM [27] and integrins α5 and β3 [28,29]. In this regard it is interesting that ARTN was co-expressed with GFRα1 or GFRα3 in only approximately 25% of cases respectively and with either GFRα1 or GFRα3 in 35.8% of cases. We previously demonstrated that ARTN was expressed in 65.4% of tumors in this cohort [5]. Thus, a significant portion of tumors express ARTN but not GFRα1 or GFRα3 suggestive that alternative receptors for ARTN may be expressed in these tumors. One other explanation is that a percentage of tumors with ARTN expression may not functionally respond to ARTN due to lack of expression of proteins binding ARTN. ARTN sensitive cancers of varying origin may also utilize different ARTN binding receptors, or different combinations thereof, to promote tumor progression. However, other reports [25] have demonstrated that the protein levels of both ARTN and GFRα3 were significantly increased in pancreatic cancer compared to normal tissue by 30-fold and 20-fold respectively indicative of potential co-ordinated increased expression although this was not specifically determined. In any case, our work herein suggests that expression of GFRα1 and/or GFRα3, especially when combined with ARTN expression, may be a useful predictor of disease progression and outcome in MC.

Previous studies have examined the expression of GFRα1 and RET in MC ([4,30]; for review see [31]) However, these studies did not examine potential correlations of the expression of GFRα1 with survival outcome nor the significance of co-expression of GFRα1 with GFRα3 nor ARTN. Concordant with our study herein, Esseghir et al. [4] reported that expression of GFRα1 mRNA was increased in MC compared with normal mammary tissue. Furthermore, and consistent with our results, higher levels of GFRα1 mRNA were reported to be associated with tumor lymphovascular invasion and lymph node metastasis [4]. However, while Esseghir et al. [4] reported that GFRα1 mRNA was associated with both ER and PR status we failed to observe such a correlation herein. The potential reasons for this discrepancy are not apparent but could be due to differences in the material investigated, differences in the visualization methods, evaluation scoring used in IHC and ISH, or the heterogeneity of the disease. The patient cohort utilized herein was entirely of Han Chinese ethnicity whereas the cohort utilized by Esseghir et al. [4] was sourced in the United Kingdom. We have however, previously described that ARTN is associated with ER status [14], despite its expression in ER negative MC, and is estrogen regulated. Furthermore, RET has been reported to be expressed preferentially in ER positive MC [32]. We have however described a clear metastasis promoting role for ARTN in ER negative MC [11] consistent with the association of GFRα1 and GFRα3 expression with lymph node metastasis observed in this study. Furthermore, significant associations of GFRα1 and GFRα3 expression with survival outcome observed herein was restricted to ER negative MC. It should be noted that autonomous expression of estrogen regulated genes are often utilized in the transition from estrogen dependent to estrogen independent growth of MC [33]. Consistent with this notion, ARTN has been reported to promote both estrogen independent growth of ER positive MC cells and resistance to anti-estrogen therapy [14].

## Conclusion

In this study, we demonstrate that expression of GFRα1 or GFRα3, particularly in combination with ARTN, is associated with worse survival outcome for patients, specifically with ER negative and HER-2 negative MC. Expression of these proteins may therefore be useful as prognostic markers in certain subtypes of MC and for selection of patients where inhibition of ARTN is to be considered as a therapeutic strategy. Whether ARTN also binds to other proteins, as yet to be identified, to mediate its effects on progression of MC remains to be determined.

## Abbreviations

ARTN: Artemin; BBD: Benign breast disease; GDNF: Glial cell line-derived neurotrophic factor; GFRα1: GDNF family receptor alpha-1; GFRα3: GDNF family receptor alpha-3; HER-2: Human epidermal growth factor receptor 2; MC: Mammary carcinoma; OS: Overall survival; RFS: Relapse-free survival; LNM: Lymph node metastasis; SDC3: Syndecan-3; TMA: Tissue microarray.

## Competing interests

PEL is an inventor on PCT/NZ2008/000152 and PCT/NZ2010/000207 and derivatives thereof. TZ and PEL previously consulted for Saratan Therapeutics Ltd. ZSW, VP, WYW and SY have nothing to declare.

## Authors’ contributions

ZSW, VP, WYW and SY performed experiments and summarized the data; ZSW, TZ and PEL designed experiments; ZSW and PEL wrote the paper; all authors have read and approved the final manuscript.

## Pre-publication history

The pre-publication history for this paper can be accessed here:

http://www.biomedcentral.com/1471-2407/13/34/prepub

## Supplementary Material

Additional file 1: Table S1 Matrix of the Spearman’s correlations between ARTN expression and either GFRα1, GFRα3 and SDC3 mRNA or protein expression in mammary carcinoma (n = 159). Table S2 Co-expression of ARTN with GFRα1, GFRα3 or SDC3 protein in mammary carcinoma patients (n = 159). Table S3 Association of tumor GFRα1, GFRα3 and SDC3 expression with five year relapse free (RFS) and overall survival (OS) in patients with mammary carcinoma. Table S4 Multivariate analysis of tumor GFRα1, GFRα3 and SDC3 expression with five year relapse free (RFS) and overall survival (OS) in patients with mammary carcinoma. Table S5 Association of tumor GFRα1, GFRα3 and SDC3 expression with five year relapse free (RFS) and overall survival (OS) in patients with ER-positive mammary carcinoma. Table S6 Association of tumor ARTN, GFRα1, GFRα3 and SDC3 expression with five year relapse free (RFS) and overall survival (OS) in patients with ER negative mammary carcinoma. Table S7 Association of tumor GFRα1, GFRα3 and SDC3 expression with five year relapse free (RFS) and overall survival (OS) in patients with HER2-negative mammary carcinoma. Table S8 Association of tumor ARTN, GFRα1, GFRα3 and SDC3 expression with five year relapse free (RFS) and overall survival (OS) in patients with HER2-positive mammary carcinoma. Table S9 Association of tumor ARTN, GFRα1, GFRα3 and SDC3 expression with five year relapse free (RFS) and overall survival (OS) in patients with mammary carcinoma. Table S10 Multivariate analysis of tumor ARTN, GFRα1, GFRα3 and SDC3 expression with five year relapse free (RFS) and overall survival (OS) in patients with mammary carcinoma. Click here for file
