# Supplementary material for: Prognostic significance of the expression of GFRα1, GFRα3 and Syndecan-3, proteins binding ARTEMIN, in mammary carcinoma
Source: BMC Cancer. 2013 Jan 26;13:34. doi: 10.1186/1471-2407-13-34 (PMC3562211; doi:10.1186/1471-2407-13-34)
Supplement: Additional file 1: Table S1 — Matrix of the Spearman’s correlations between ARTN expression and either GFRα1, GFRα3 and SDC3 mRNA or protein expression in mammary carcinoma (n = 159). Table S2 Co-expression of ARTN with GFRα1, GFRα3 or SDC3 protein in mammary carcinoma patients (n = 159). Table S3 Association of tumor GFRα1, GFRα3 and SDC3 expression with five year relapse free (RFS) and overall survival (OS) in patients with mammary carcinoma. Table S4 Multivariate analysis of tumor GFRα1, GFRα3 and SDC3 expression with five year relapse free (RFS) and overall survival (OS) in patients with mammary carcinoma. Table S5 Association of tumor GFRα1, GFRα3 and SDC3 expression with five year relapse free (RFS) and overall survival (OS) in patients with ER-positive mammary carcinoma. Table S6 Association of tumor ARTN, GFRα1, GFRα3 and SDC3 expression with five year relapse free (RFS) and overall survival (OS) in patients with ER negative mammary carcinoma. Table S7 Association of tumor GFRα1, GFRα3 and SDC3 expression with five year relapse free (RFS) and overall survival (OS) in patients with HER2-negative mammary carcinoma. Table S8 Association of tumor ARTN, GFRα1, GFRα3 and SDC3 expression with five year relapse free (RFS) and overall survival (OS) in patients with HER2-positive mammary carcinoma. Table S9 Association of tumor ARTN, GFRα1, GFRα3 and SDC3 expression with five year relapse free (RFS) and overall survival (OS) in patients with mammary carcinoma. Table S10 Multivariate analysis of tumor ARTN, GFRα1, GFRα3 and SDC3 expression with five year relapse free (RFS) and overall survival (OS) in patients with mammary carcinoma. [file 1471-2407-13-34-S1.doc]

**Supplemental table 1.** Matrix of the Spearman’s correlations between ARTN expression and either GFRα1, GFRα3 and SDC3 mRNA or protein expression in mammary carcinoma (n=159).

|  |  |  | ARTN protein |  | GFRα1 mRNA |  | GFRα3 mRNA |  | SDC3 mRNA |
| --- | --- | --- | --- | --- | --- | --- | --- | --- | --- |
| ARTN protein | r_s_ |  |  |  | 0.176 |  | 0.361 |  | 0.13 |
|  | *P* |  |  |  | **0.026** |  | **<0.001** |  | 0.102 |
| GFRα1 protein | r_s_ |  | 0.13 |  | 0.514 |  | 0.478 |  | 0.008 |
|  | *P* |  | 0.103 |  | **<0.001** |  | **<0.001** |  | 0.916 |
| GFRα3 protein | r_s_ |  | 0.208 |  | 0.483 |  | 0.619 |  | 0.149 |
|  | *P* |  | **0.009** |  | **<0.001** |  | **<0.001** |  | 0.061 |
| SDC3 protein | r_s_ |  | 0.134 |  | 0.066 |  | 0.133 |  | 0.287 |
|  | *P* |  | 0.092 |  | 0.409 |  | 0.091 |  | **<0.001** |
|  |  |  |  |  |  |  |  |  |  |

Values in *bold* are significant (*P* < 0.05).

**Supplemental table 2** Co-expression of ARTN with GFRα1, GFRα3 or SDC3 protein in mammary carcinoma patients (n=159)

|  |  | Positive expression (n (%)) | | | | |
| --- | --- | --- | --- | --- | --- | --- |
|  |  | GFRα1 | GFRα3 | GFRα1 or GFRα3 | SDC3 | GFRα1 or GFRα3 or SD3 |
| ARTN- |  | 16 (10.1) | 10 (6.3) | 21 (13.2) | 11 (6.9) | 27 (17) |
| ARTN+ |  | 44 (27.7) | 40 (25.2) | 57 (35.8) | 34 (21.4) | 73 (45.9) |

**Supplemental table 3.** Association of tumor GFRα1, GFRα3 and SDC3 expression with five year relapse free (RFS) and overall survival (OS) in patients with mammary carcinoma.

|  | RFS (%) | | | |  | OS (%) | | | |
| --- | --- | --- | --- | --- | --- | --- | --- | --- | --- |
|  | mRNA | *P* | protein | *P* |  | mRNA | *P* | protein | *P* |
| GFRα1-/GFRα1+ | 66.1*/*50.7 | 0.075 | 68.0*/*43.1 | **0.003** |  | 69.5*/*58.2 | 0.155 | 73.3*/*49.0 | **0.004** |
| GFRα3-/GFRα3+ | 66.7*/*45.1 | **0.008** | 67.1*/*39.0 | **0.002** |  | 70.7*/*52.9 | **0.03** | 70.6*/*48.8 | **0.011** |
| SDC3-*/*SDC3+ | 51.7*/*71.8 | 0.07 | 57.6*/*58.8 | 0.856 |  | 58.6*/*74.4 | 0.105 | 60.9*/*70.6 | 0.359 |
| GFRα1-GFRα3-/GFRα1+GFRα3+ | 70.4*/*47.8 | **0.017** | 71.9*/*36.7 | **0.001** |  | 74.1*/*56.5 | **0.048** | 76.6*/*46.7 | **0.002** |
| GFRα1- SDC3-/GFRα1+SDC3+ | 64.3*/*72.7 | 0.557 | 62.7*/*47.1 | 0.102 |  | 69.0*/*77.3 | 0.523 | 69.0*/*52.9 | 0.186 |
| GFRα3-SDC3-/GFRα3+SDC3+ | 62.5*/*65.0 | 0.948 | 67.2*/*46.2 | 0.09 |  | 67.9*/*70.0 | 0.926 | 67.2*/*53.8 | 0.248 |
| GFRα1-SDC3-GFRα3-/GFRα1-SDC3-GFRα3+ | 69.2*/*0 | **0.002** | 72.0*/*37.5 | 0.117 |  | 74.4*/*0 | **0.001** | 72.0*/*50.0 | 0.318 |
| GFRα3-SDC3-GFRα1-/GFRα3-SDC3-GFRα1+ | 69.2*/*47.1 | 0.13 | 72.0*/*50.0 | 0.18 |  | 74.4*/*52.9 | 0.083 | 72.0*/*50.0 | 0.139 |

Values in *bold* are significant (*P* < 0.05).

**Supplemental table 4.** Multivariate analysis of tumor GFRα1, GFRα3 and SDC3 expression with five year relapse free (RFS) and overall survival (OS) in patients with mammary carcinoma.

|  | RFS -Odds ratio (95% CI) | | | |  | OS-Odds ratio (95% CI) | | | |
| --- | --- | --- | --- | --- | --- | --- | --- | --- | --- |
|  | mRNA | *P* | protein | *P* |  | mRNA | *P* | protein | *P* |
| GFRα1-/GFRα1+ | 1.639 (0.94-2.856) | 0.081 | 2.200 (1.279-3.784) | **0.004** |  | 1.526 (0.844-2.76) | 0.162 | 2.285 (1.274-4.099) | **0.006** |
| GFRα3-/GFRα3+ | 2.055 (1.191-3.547) | **0.01** | 2.308 (1.345-3.961) | **0.002** |  | 1.869 (1.048-3.335) | **0.034** | 2.077 (1.162-3.714) | **0.014** |
| SDC3-*/*SDC3+ | 0.548 (0.282-1.067) | 0.077 | 0.946 (0.513-1.742) | 0.858 |  | 0.568 (0.282-1.144) | 0.113 | 0.724 (0.359-1.459) | 0.366 |
| GFRα1-GFRα3-/GFRα1+GFRα3+ | 2.106 (1.118-3.967) | **0.021** | 3.024 (1.584-5.773) | **0.01** |  | 1.960 (0.990-3.882) | 0.054 | 2.850 (1.406-5.777) | **0.004** |
| GFRα1- SDC3-/GFRα1+SDC3+ | 0.755 (0.293-1.946) | 0.56 | 1.909 (0.863-4.222) | 0.111 |  | 0.716 (0.255-2.009) | 0.526 | 1.734 (0.753-3.99) | 0.196 |
| GFRα3- SDC3-/GFRα3+SDC3+ | 1.029 (0.435-2.434) | 0.949 | 2.052 (0.87-4.841) | 0.101 |  | 0.957 (0.38-2.412) | 0.926 | 1.688 (0.681-4.185) | 0.259 |
| GFRα1-SDC3-GFRα3-/GFRα1-SDC3-GFRα3+ | 6.125 (1.631-23.002) | **0.007** | 2.206 (0.793-6.133) | 0.129 |  | 1.45 (1.03-2.05) | **0.035** | 1.744 (0.573-5.302) | 0.327 |
| GFRα3-SDC3-GFRα1-/GFRα3-SDC3-GFRα1+ | 1.929 (0.807-4.607) | 0.139 | 1.828 (0.737-4.532) | 0.193 |  | 2.223 (0.875-5.643) | 0.093 | 1.949 (0.785-4.841) | 0.15 |

Values in *bold* are significant (*P* < 0.05).

**Supplemental table 5.** Association of tumor GFRα1, GFRα3 and SDC3 expression with five year relapse free (RFS) and overall survival (OS) in patients with ER-positive mammary carcinoma.

|  | RFS (%) | | | |  | OS (%) | | | |
| --- | --- | --- | --- | --- | --- | --- | --- | --- | --- |
|  | mRNA | *P* | protein | *P* |  | mRNA | *P* | protein | *P* |
| GFRα1-/GFRα1+ | 60.0/60.0 | 0.807 | 68.8/44.4 | 0.095 |  | 64.0/76.0 | 0.369 | 75.0/61.1 | 0.271 |
| GFRα3-/GFRα3+ | 65.5/52.4 | 0.249 | 69.7/41.2 | 0.091 |  | 72.4/ 66.7 | 0.597 | 72.7/64.7 | 0.562 |
| SDC3-/SDC3+ | 50.0/77.8 | 0.104 | 51.4/80.0 | 0.052 |  | 62.5/83.3 | 0.143 | 60.0/93.3 | **0.023** |
| GFRα1-GFRα3-/GFRα1+GFRα3+ | 63.6*/*55.6 | 0.676 | 73.1*/*36.4 | **0.048** |  | 68.2*/*72.2 | 0.831 | 76.9*/*63.6 | 0.376 |
| GFRα1- SDC3-/GFRα1+SDC3+ | 58.8*/*90.0 | 0.082 | 59.1*/*60.0 | 0.784 |  | 64.7*/*100 | **0.041** | 63.6*/*80.0 | 0.494 |
| GFRα3- SDC3-/GFRα3+SDC3+ | 66.7*/*90.0 | 0.234 | 63.6*/*75.0 | 0.517 |  | 76.2*/*100 | 0.104 | 63.6*/*100 | 0.183 |
| GFRα1-SDC3-GFRα3-/GFRα1-SDC3-GFRα3+ | 66.7*/*0 | **0.001** | 66.7*/*50.0 | 0.682 |  | 73.3*/*0 | **0.002** | 66.7*/*50.0 | 0.736 |
| GFRα3-SDC3-GFRα1-/GFRα3-SDC3-GFRα1+ | 66.7*/*66.7 | 0.678 | 72.0*/*50.0 | 0.18 |  | 73.3*/* 83.3 | 0.642 | 66.7*/*50.0 | 0.632 |

Values in *bold* are significant (*P* < 0.05).

**Supplemental table 6.** Association of tumor ARTN, GFRα1, GFRα3 and SDC3 expression with five year relapse free (RFS) and overall survival (OS) in patients with ER negative mammary carcinoma.

|  | RFS (%) | | | |  | OS (%) | | | |
| --- | --- | --- | --- | --- | --- | --- | --- | --- | --- |
|  | mRNA | *P* | protein | *P* |  | mRNA | *P* | protein | *P* |
| GFRα1-/GFRα1+ | 70.6/45.2 | **0.02** | 67.4/42.4 | **0.022** |  | 73.5/47.6 | **0.018** | 72.1/42.4 | **0.009** |
| GFRα3-/GFRα3+ | 67.4/40.0 | **0.012** | 65.4/37.5 | **0.006** |  | 69.6/43.3 | **0.017** | 69.2/37.5 | **0.004** |
| SDC3-/SDC3+ | 52.7/66.7 | 0.35 | 61.4/42.1 | 0.143 |  | 56.4/66.7 | 0.424 | 60.0/93.3 | 0.433 |
| GFRα1-GFRα3-/GFRα1+GFRα3+ | 75.0*/*42.9 | **0.008** | 71.1*/*36.8 | **0.004** |  | 78.1*/*46.4 | **0.008** | 76.3*/*36.8 | **0.002** |
| GFRα1- SDC3-/GFRα1+SDC3+ | 68.0*/*58.3 | 0.476 | 72.2*/*41.7 | **0.035** |  | 72.0*/*58.3 | 0.376 | 72.2*/*41.7 | **0.039** |
| GFRα3- SDC3-/GFRα3+SDC3+ | 60.0*/*40.0 | 0.153 | 69.0*/*33.3 | **0.008** |  | 62.9*/*40.0 | 0.157 | 69.0*/*33.3 | **0.012** |
| GFRα1-SDC3-GFRα3-/GFRα1-SDC3-GFRα3+ | 70.8*/*0 | 0.212 | 75.0*/*50.0 | 0.334 |  | 75.0*/*0 | 0.142 | 75.0*/*50.0 | 0.334 |
| GFRα3-SDC3-GFRα1-/GFRα3-SDC3-GFRα1+ | 70.8*/*36.4 | **0.038** | 75.0*/*50.0 | 0.177 |  | 75.0*/* 36.4 | **0.018** | 75.0*/*50.0 | 0.141 |

Values in *bold* are significant (*P* < 0.05).

**Supplemental table 7.** Association of tumor GFRα1, GFRα3 and SDC3 expression with five year relapse free (RFS) and overall survival (OS) in patients with HER2-negative mammary carcinoma.

|  | RFS (%) | | | |  | OS (%) | | | |
| --- | --- | --- | --- | --- | --- | --- | --- | --- | --- |
|  | mRNA | *P* | protein | *P* |  | mRNA | *P* | protein | *P* |
| GFRα1-/GFRα1+ | 66.0/47.6 | 0.079 | 68.8/35.5 | **0.002** |  | 69.8/50.0 | 0.175 | 73.4/45.2 | **0.005** |
| GFRα3-/GFRα3+ | 65.6/44.1 | **0.024** | 67.1/32.0 | **0.003** |  | 70.5/ 52.9 | 0.067 | 70.0/48.0 | **0.042** |
| SDC3-/SDC3+ | 50.0/75.9 | **0.045** | 56.3/62.5 | 0.55 |  | 57.6/79.3 | 0.068 | 60.6/75.0 | 0.234 |
| GFRα1-GFRα3-/GFRα1+GFRα3+ | 70.8*/*48.3 | **0.043** | 74.5*/*31.3 | **0.001** |  | 75.0*/*58.6 | 0.108 | 78.2*/*50.0 | **0.019** |
| GFRα1- SDC3-/GFRα1+SDC3+ | 63.2*/*78.6 | 0.327 | 67.3*/*44.4 | 0.215 |  | 68.4*/*85.7 | 0.261 | 69.4*/*55.6 | 0.398 |
| GFRα3- SDC3-/GFRα3+SDC3+ | 60.9*/*71.4 | 0.629 | 66.0*/*42.9 | 0.268 |  | 67.4*/*78.6 | 0.521 | 66.0*/*57.1 | 0.59 |
| GFRα1-SDC3-GFRα3-/GFRα1-SDC3-GFRα3+ | 68.6*/*0 | **0.002** | 74.4*/*16.7 | **0.014** |  | 74.3*/*0 | **0.001** | 74.4*/*33.3 | 0.071 |
| GFRα3-SDC3-GFRα1-/GFRα3-SDC3-GFRα1+ | 68.6*/*36.4 | 0.068 | 74.4*/*30.0 | **0.012** |  | 74.3*/*45.5 | **0.044** | 74.4*/*30.0 | **0.007** |

Values in *bold* are significant (*P* < 0.05).

**Supplemental table 8**. Association of tumor ARTN, GFRα1, GFRα3 and SDC3 expression with five year relapse free (RFS) and overall survival (OS) in patients with HER2-positive mammary carcinoma.

|  | RFS (%) | | | |  | OS (%) | | | |
| --- | --- | --- | --- | --- | --- | --- | --- | --- | --- |
|  | mRNA | *P* | protein | *P* |  | mRNA | *P* | protein | *P* |
| GFRα1-/GFRα1+ | 66.7/56.0 | 0.608 | 63.6/55.0 | 0.522 |  | 66.7/60.0 | 0.701 | 72.7/55.0 | 0.343 |
| GFRα3-/GFRα3+ | 71.4/47.1 | 0.16 | 66.7/50.0 | 0.23 |  | 71.4/52.9 | 0.287 | 73.3/50.0 | 0.145 |
| SDC3-/SDC3+ | 57.1/60.0 | 0.892 | 61.9/50.0 | 0.526 |  | 61.9/60.0 | 0.922 | 61.9/60.0 | 0.89 |
| GFRα1-GFRα3-/GFRα1+GFRα3+ | 66.7*/*47.1 | 0.413 | 55.6*/*42.9 | 0.376 |  | 66.7*/*52.9 | 0.524 | 66.7*/*42.9 | 0.257 |
| GFRα1- SDC3-/GFRα1+SDC3+ | 75.0*/*62.5 | 0.653 | 66.7*/*50.0 | 0.398 |  | 75.0*/*62.5 | 0.726 | 66.7*/*50.0 | 0.478 |
| GFRα3- SDC3-/GFRα3+SDC3+ | 70.0*/*50.0 | 0.367 | 72.7*/*50.0 | 0.193 |  | 70.0*/*50.0 | 0.515 | 72.7*/*50.0 | 0.272 |
| GFRα1-SDC3-GFRα3-/GFRα1-SDC3-GFRα3+ | NA |  | 57.1*/*100.0 | 0.305 |  | NA |  | 57.1*/*100.0 | 0.313 |
| GFRα3-SDC3-GFRα1-/GFRα3-SDC3-GFRα1+ | 75.0*/*66.7 | 0.83 | 57.1*/*100.0 | 0.15 |  | 75.0*/*66.7 | 0.796 | 57.1*/*100.0 | 0.154 |

Note: NA = not available.

**Supplemental table 9.** Association of tumor ARTN, GFRα1, GFRα3 and SDC3 expression with five year relapse free (RFS) and overall survival (OS) in patients with mammary carcinoma.

|  | RFS (%) | |  | OS (%) | |
| --- | --- | --- | --- | --- | --- |
|  | protein | *P* |  | protein | *P* |
| ARTN-/ARTN+ | 62.5*/*47.4 | 0.083 |  | 70.5*/*47.4 | **0.021** |
| ARTN-GFRα1-/ARTN+GFRα1+ | 78.3*/*30.0 | **0.001** |  | 87.0*/*30.0 | **0.001** |
| ARTN-GFRα1+/ARTN+GFRα1+ | 46.3*/*30.0 | 0.334 |  | 53.7*/*30.0 | 0.240 |
| ARTN+GFRα1-/ARTN+GFRα1+ | 53.6*/*30.0 | 0.193 |  | 53.6*/*30.0 | 0.159 |
| ARTN-GFRα3-/ARTN+GFRα3+ | 77.8*/*37.5 | **0.009** |  | 83.3*/*37.5 | **0.003** |
| ARTN-GFRα3+/ARTN+GFRα3+ | 39.4*/*37.5 | 0.975 |  | 51.5*/*37.5 | 0.803 |
| ARTN+GFRα3-/ARTN+GFRα3+ | 50. 0*/*37.5 | 0.835 |  | 50. 0*/*37.5 | 0.747 |
| ARTN-SDC3-*/*ARTN*+*SDC3+ | 64.4*/*60.0 | 0.88 |  | 69.5*/*60.0 | 0.755 |

Values in *bold* are significant (*P* < 0.05).

**Supplemental table 10.** Multivariate analysis of tumor ARTN, GFRα1, GFRα3 and SDC3 expression with five year relapse free (RFS) and overall survival (OS) in patients with mammary carcinoma.

|  | RFS -Odds ratio (95% CI) | |  | OS-Odds ratio (95% CI) | |
| --- | --- | --- | --- | --- | --- |
|  | protein | *P* |  | protein | *P* |
| ARTN-*/*ARTN*+* | 1.625 (0.928-2.845) | 0.089 |  | 1.949 (1.087-3.493) | **0.025** |
| ARTN-GFRα1-/ARTN+GFRα1+ | 4.812 (1.799-12.874) | **0.002** |  | 6.942 (2.317-20.796) | **0.001** |
| ARTN-GFRα1+/ARTN+GFRα1+ | 1.514 (0.640-3.579) | 0.345 |  | 1.664 (0.697-3.974) | 0.251 |
| ARTN+GFRα1-/ARTN+GFRα1+ | 1.347 (0.848-2.140) | 0.207 |  | 1.382 (0.868-2.200) | 0.172 |
| ARTN-GFRα3-/ARTN+GFRα3+ | 3.684 (1.29-10.523) | **0.015** |  | 4.501 (1.503-13.478) | **0.007** |
| ARTN-GFRα3+/ARTN+GFRα3+ | 0.984 (1.367-2.641) | 0.975 |  | 1.136 (0.416-3.102) | 0.804 |
| ARTN+GFRα3-/ARTN+GFRα3+ | 1.054 (0.635-1.750) | 0.838 |  | 1.085 (0.654-1.801) | 0.751 |
| ARTN- SDC3-*/*ARTN+SDC3+ | 1.117 (0.262-4.766) | 0.882 |  | 1.258 (0.292-5.425) | 0.758 |

Values in *bold* are significant (*P* < 0.05).
